# Correlates of long-term clinical outcomes in pediatric multiple sclerosis: A 12-year study

**DOI:** 10.1177/13524585251408639

**Published:** 2026-01-26

**Authors:** Monica Margoni, Alessandro Meani, Elisabetta Pagani, Paolo Preziosa, Lucia Moiola, Mattia Pozzato, Eleonora Tavazzi, Flavia Mattioli, Valentina Torri Clerici, Massimo Filippi, Maria A Rocca

**Affiliations:** Neuroimaging Research Unit, Division of Neuroscience, IRCCS San Raffaele Scientific Institute, Milan, Italy; Neurology Unit, IRCCS San Raffaele Scientific Institute, Milan, Italy; Neurorehabilitation Unit, IRCCS San Raffaele Scientific Institute, Milan, Italy; Neuroimaging Research Unit, Division of Neuroscience, IRCCS San Raffaele Scientific Institute, Milan, Italy; Neuroimaging Research Unit, Division of Neuroscience, IRCCS San Raffaele Scientific Institute, Milan, Italy; Neuroimaging Research Unit, Division of Neuroscience, IRCCS San Raffaele Scientific Institute, Milan, Italy; Neurology Unit, IRCCS San Raffaele Scientific Institute, Milan, Italy; Vita-Salute San Raffaele University, Milan, Italy; Neurology Unit, IRCCS San Raffaele Scientific Institute, Milan, Italy; Neuroimmunology Unit, Multiple Sclerosis Centre, ASST Valle Olona, Gallarate, Italy; Multiple Sclerosis Centre, IRCCS Mondino Foundation, Pavia, Italy; Neuropsychology Unit, Spedali Civili of Brescia, Brescia, Italy; Fondazione IRCCS Istituto Neurologico “C. Besta”, U.O. Neuroimmunologia e Malattie Neuromuscolari, Milan, Italy; Neuroimaging Research Unit, Division of Neuroscience, IRCCS San Raffaele Scientific Institute, Milan, Italy; Neurology Unit, IRCCS San Raffaele Scientific Institute, Milan, Italy; Neurorehabilitation Unit, IRCCS San Raffaele Scientific Institute, Milan, Italy; Vita-Salute San Raffaele University, Milan, Italy; Neurophysiology Service, IRCCS San Raffaele Scientific Institute, Milan, Italy; Neuroimaging Research Unit, Division of Neuroscience, IRCCS San Raffaele Scientific Institute, Milan, Italy; Neurology Unit, IRCCS San Raffaele Scientific Institute, Milan, Italy; Vita-Salute San Raffaele University, Milan, Italy

**Keywords:** Pediatric multiple sclerosis, MRI, correlates, relapse, disability worsening

## Abstract

**Background:**

Identifying long-term outcome substrates in pediatric multiple sclerosis (Ped-MS) can inform treatment selection. We investigated clinical/magnetic resonance imaging (MRI) correlates over a median 12.3-year follow-up.

**Methods:**

Fifty-two Ped-MS patients and 23 healthy controls underwent baseline 3.0 T MRI. Neurological evaluations were performed every 6 months. MRI metrics included regional lesion burden, brain and choroid plexus (CP) volumes, diffusion tensor-derived fractional anisotropy (FA), averaged in normal-appearing white matter (NAWM). Multivariable Cox, Andersen–Gill, linear regression models identified long-term outcome correlates.

**Results:**

At follow-up, 69% relapsed, 21% had 6-month confirmed disability worsening (6m-CDW), and 35% showed EDSS worsening. Infratentorial lesion number (hazard ratio (HR) = 1.09, 95% confidence interval (CI) = 1.02 to 1.18), spinal cord lesion presence (HR = 2.45, 95% CI = 1.18 to 5.10), and thalamic volume (HR = 0.77, 95% CI = 0.60 to 0.99) were associated with shorter time to first relapse; high-efficacy treatment (HET) exposure with longer (HR = 0.20, 95% CI = 0.06 to 0.68), and CP volume (HR = 1.58, 95% CI = 0.93 to 2.68) was associated marginally. WM lesion volume (HR = 1.04, 95% CI = 1.01 to 1.06) and HET exposure (HR = 0.21, 95% CI = 0.11 to 0.40) were associated with higher/lower overall relapse risk, respectively; baseline EDSS (HR = 1.29, 95% CI = 0.96 to 1.73), spinal cord lesion presence (HR = 1.52, 95% CI = 0.95 to 2.44), and CP volume (HR = 1.35, 95% CI = 1.00 to 1.83) contributed marginally. Younger age (HR = 0.82, 95% CI = 0.68 to 0.98) and NAWM FA (HR = 0.67, 95% CI = 0.51 to 0.88) were associated with earlier 6m-CDW; NAWM FA with EDSS worsening (β = −0.26, 95% CI = −0.50 to −0.03).

**Conclusion:**

Advanced MRI markers and HET exposure associate with long-term outcomes in Ped-MS.

## Introduction

Pediatric-onset multiple sclerosis (MS) accounts for about 2% to 10% of the total MS cases.^1^ Pediatric multiple sclerosis (Ped-MS) patients typically exhibit higher relapse rate and accrual of brain white matter (WM) lesions early in their disease course, but they have better clinical recovery compared with adult-onset MS,^2^ likely due to age-related brain repair and plasticity,^3^ which may limit long-term disability.^2^ Recent evidence suggests that smoldering processes start from disease onset,^4,5^ making it crucial to identify correlates of long-term outcomes in these patients to guide treatment selection.

Only a few longitudinal studies have assessed the substrates of relapses and disability in Ped-MS patients using clinical or conventional magnetic resonance imaging (MRI) features.^2,6–8^ Female sex, older age at onset, multifocal onset, and optic nerve involvement were associated with second clinical attacks in pediatric patients with a clinically isolated syndrome.^6–8^ Conversely, older age at onset, multifocal and/or spinal cord/infratentorial involvement, higher relapse rate, and T2-hyperintense WM lesions were associated with disability progression over 9 years.^6–8^

Considering the heterogeneous pathological processes in Ped-MS, advanced MRI techniques may provide more specific insights than conventional MRI. MRI protocols should be optimized to minimize scanning time while maintaining high image quality, thereby enhancing clinical feasibility and providing meaningful additional information. In adult MS, the location of focal lesions in cortical,^9,10^ infratentorial,^11^ and spinal cord areas^12^ was consistently associated with future disability over 5–30 years. Thalamic and cortical atrophy,^13,14^ along with normal-appearing (NA) WM microstructural abnormalities,^15^ correlated with disability progression up to 15 years. Recently, enlarged choroid plexus (CP) volume has been described from the earliest phases of MS, even in pediatric patients,^16,17^ being associated with future relapses^18^ and disability.^19^ Integrating advanced MRI markers with clinical and conventional measures may improve prognostic accuracy and inform tailored intervention in Ped-MS. Against this background, this study aimed to identify clinical and MRI correlates of long-term clinical outcomes in Ped-MS patients, integrating advanced MRI markers with clinical and conventional lesional measures. Specifically, we evaluated lesion distribution in typical brain and spinal cord regions, brain and CP volumetric measures, and normal-appearing white matter (NAWM) integrity using diffusion tensor (DT) MRI. Then, we assessed their associations with clinical outcomes: time to first relapse, risk of relapse occurrence, time to 6-month confirmed disability worsening (6m-CDW), and Expanded Disability Status Scale (EDSS) worsening at last follow-up.

## Methods

### Participants

In this longitudinal, observational, multicenter study, we included 70 consecutive relapsing-remitting (RR) pediatric-onset MS patients, diagnosed according to the 2017 McDonald criteria, recruited from November 2008 to January 2024. Diagnoses were retrospectively reviewed to ensure updated criteria were met. Patients had to be relapse- and steroid-free for at least 1 month prior to clinical and MRI assessment. Whenever needed, appropriate testing was performed to exclude myelin oligodendrocyte glycoprotein antibody-associated disease (MOGAD) (e.g. individuals younger than 12 years and/or presenting with atypical clinical and radiological findings). Exclusion criteria were a history of other primary neurological or psychiatric disorders in addition to MS. Twenty-three healthy controls (HCs) with no previous history of neurological dysfunction and a normal neurological examination performed by an experienced neurologist served as the control group. Pediatric healthy subjects were mainly recruited among children of healthcare personnel and by word of mouth.

### Clinical assessment

At baseline, Ped-MS patients underwent neurologic evaluation, with EDSS score rating and recording of ongoing disease-modifying treatments (DMTs) on the day of MRI acquisition. Longitudinal neurological visits were conducted at least every 6 months, documenting clinical phenotype, EDSS score, relapses, and ongoing DMTs. 6m-CDW was defined as 1.5-point increase (with baseline EDSS score = 0.0), 1.0-point increase (with baseline EDSS score < 5.5), or 0.5-point increase (with baseline EDSS score ⩾ 5.5), confirmed 6 months apart. DMTs were grouped into moderate-efficacy treatment (MET: interferon-beta, glatiramer acetate, teriflunomide, and dimethyl fumarate) and high-efficacy treatment (HET: cyclophosphamide, mitoxantrone, cladribine, natalizumab, fingolimod, alemtuzumab, ocrelizumab).

### MRI acquisition

Baseline brain MRI was performed on a 3.0 T Philips Intera scanner with an 8-channel coil. Sequences included (1) dual-echo turbo spin echo; (2) three-dimensional (3D) T1-weighted fast field echo; (3) 3D double inversion recovery (DIR); and (4) pulsed-gradient spin echo echo-planar imaging with SENSE (acceleration factor = 2) and diffusion gradients applied in 35 non-collinear directions (exam session duration: 34 min) (see Supplementary Methods for additional details on the MRI protocol).

### Lesional and volumetric MRI analysis

T2-hyperintense WM lesion volume (LV) was quantified on the dual-echo sequence using a local thresholding segmentation technique (Jim 8, Xinapse Systems). Cortical lesions were manually identified on DIR images by two raters (P.P. and M.M.) following published recommendations;^20^ disagreements were resolved by a third rater (M.A.R.).

Lesion topography was defined as infratentorial, periventricular, juxtacortical, or deep WM based on co-registration with anatomical masks (see Supplementary Methods for additional details). For each class we counted the total volume of T2-hyperintense WM lesions assigned. Spinal cord lesion presence was assessed retrospectively from spinal MRI acquired closely to baseline MRI, performed in all patients during the diagnostic work-up and repeated if new spinal symptoms occurred.

Normalized brain (NBV), cortical volume (NCV), and thalamic and ventricular volumes were measured on the 3D T1-weighted sequence using the FSL-SIENA×2 software, after lesion refilling. Lateral ventricle volume was then measured after manual editing of the ventricular cerebrospinal fluid (CSF) map.

### DT MRI analysis

Diffusion-weighted images were corrected for off-resonance, eddy current-induced distortions, and movements using the Eddy tool in the FSL library. Because reversed-gradient b0 was unavailable, an undistorted b0 was simulated from 3D T1-weighted sequence for distortion correction. The DT was estimated by linear regression, and fractional anisotropy (FA), mean (MD), axial (AD), and radial diffusivity (RD) maps were derived. T2-hyperintense lesions were excluded from the WM mask to derive NAWM microstructural measures (see Supplementary Methods for details).

### Segmentation and quantification of CP volume

The CP of the lateral ventricles was manually segmented on the 3D T1-weighted sequence using a local thresholding segmentation technique (Jim 8, Xinapse Systems), and then their volume was calculated.^16^ Volumes were normalized for head size using SIENA×2 scaling.

## Statistical analysis

Prior to statistical analyses, normality of all continuous variables was assessed using the Shapiro–Wilk test and visual inspection with histograms and Q–Q plots. Demographic variables were compared between Ped-MS patients and HC using Fisher’s exact and Mann–Whitney *U*-tests. Baseline clinical features were summarized using percentages for categorical and medians with quartiles for continuous variables. Differences in MRI measures were assessed by sex- and age-adjusted linear models for heteroscedastic data. Model assumptions were evaluated by inspecting residuals, including checks for heteroscedasticity and approximate normality, to ensure valid inference. Brain T2-hyperintense WM lesions were log-transformed to reduce skewness. CP volume analysis was additionally adjusted for normalized brain and lateral ventricle volumes. Although these two measures are correlated, they capture complementary aspects of brain atrophy: normalized brain volume accounts for global parenchymal loss, whereas lateral ventricle volume better reflects regional atrophy involving deep gray matter (GM) structures, which are particularly relevant in Ped-MS. This dual normalization strategy has also been previously applied^16^ and was adopted here to provide a more comprehensive adjustment of CP size. Collinearity diagnostics confirmed that their simultaneous inclusion in the model did not introduce instability. *p*-values < 0.05 were deemed statistically significant.

Estimates of the proportion of patients without clinical relapses and 6m-CDW over time were obtained using the product-limit approach. We described recurrent relapses by the mean cumulative function, representing the average cumulative number of events experienced by subjects during follow-up. Univariable extended Cox regression models were run to investigate the association of baseline demographic, clinical, and MRI variables with time to first clinical relapse and first 6m-CDW event. We studied the association with the risk of recurrent relapses using Andersen–Gill models with robust standard errors. Treatment status was included as a time-varying covariate in all the aforesaid analyses. The proportional hazards assumption was assessed based on the Schoenfeld residuals. We explored the relationships between baseline characteristics and EDSS worsening at follow-up by linear regression models, adjusting for follow-up duration, baseline treatment, and treatment change. In all our models, treatment-related covariates were included to account for heterogeneity in treatment exposure over time, capturing the real-world clinical course rather than implying causal effects. For each clinical outcome, we screened baseline demographic, clinical, and MRI features as independent factors using a stepwise (combination of forward and backward procedure) variable selection approach (*p*-value for inclusion = 0.1).

R software (version 4.3.3; “nlme,” “survival,” and “reda” packages) was used for computations.

## Results

### Demographic, clinical, and MRI variables in Ped-MS patients compared to HC

Seventy-two patients with acquired demyelinating disorders of the central nervous system were initially identified. Of these, 2 (3%) patients tested positive for MOG antibody and were therefore excluded from the study. Eighteen (25%) patients were excluded due to poor MRI image quality (*n* = 2) or loss at follow-up (*n* = 16; change of MS center = 12, living overseas = 2, no further information available = 2). Consequently, 52 Ped-MS patients were included in the final analysis. At baseline, all patients were treated with a DMT (43 patients (83%) with METs, 9 (17%) with HETs) (Table 1). At baseline, no patient was treated with fingolimod.

**Table 1.** Clinical and MRI characteristics of healthy controls and pediatric multiple sclerosis patients at baseline.

Variable		HC (*n* = 23)	Pediatric MS (*n* = 52)	*p*
Sex, No. male (%)/female (%)		13 (56)/10 (44)	19 (37)/33 (63)	0.132
Median age at baseline (IQR) (years)		14.1 (11.8 to 16.2)	15.1 (14.0 to 16.7)	0.155
Median age at disease onset (IQR) (years)		-	13.5 (11.4 to 15.0)	-
Median disease duration at baseline (IQR) (years)		-	1.4 (0.5 to 2.7)	
Type of onset				
Monofocal, No. (%)			42 (81)	
Optic neuritis			4 (10)	
Hemispheric			9 (21)	-
Infratentorial		-	20 (48)	
Spinal			9 (21)	
Multifocal (%)			10 (19)	
Median EDSS score at baseline (IQR)		-	1.5 (1.0 to 1.5)	-
No. of patients with oligoclonal bands (%)		-	52 (100)	-
Median number of previous relapses (IQR)		-	1 (1 to 2)	-
Median follow-up duration (IQR) (years)		-	12.3 (10.1 to 14.3)	-
Median EDSS score at last FU (IQR)		-	1.0 (0.0 to 2.0)	
No. of patients with EDSS worsening at last FU (%)		-	18 (35)	-
No. of patients with 6m-CDW during FU (%)			11 (21)	
No. of patients with ⩾1 clinical relapse during FU (%)		-	36 (69)	-
Estimated ARR during FU (95% CI)				
Overall			0.13 (0.10 to 0.17)	-
MET		-	0.18 (0.13 to 0.25)	-
HET			0.07 (0.04 to 0.12)	
Rate ratio HET vs. MET			0.37 (0.19 to 0.74)	0.005
Median time from disease onset to DMT start (IQR) (years)		-	0.72 (0.23 to 2.12)	-
Median DMT duration before baseline (IQR) (years)		-	0.50 (0.25 to 1.37)	-
DMTs at baseline (%): MET/HET		-	43 (83) / 9 (17)	-
No. of switchers to HET (%)		-	25/43 (58)	-
Median brain T2-hyperintense WM lesion volume (IQR) (mL)^a^		0.0 (0.0 to 0.1)	3.2 (1.6 to 6.6)	< 0.001
Median number of T2-hyperintense WM lesions (IQR)	Periventricular	-	9 (5 to 15)	-
	Juxtacortical	-	13 (6 to 34)	-
	Infratentorial	-	2 (1 to 6)	-
	Deep WM	-	5 (2 to 8)	-
Median number of cortical lesions (IQR)		-	0 (0 to 1)	
No. of patients with ⩾1 spinal cord lesion (%)		-	29 (56)	-
Estimated mean	NBV (SE) (mL)	1717 (7)	1687 (8)	0.004
	NCV (SE) (mL)	731 (7)	706 (5)	0.008
	Normalized thalamic volume (SE) (mL)	23.0 (0.2)	22.2 (0.2)	0.023
	Normalized CP volume (SE) (mL)	2.14 (0.09)	2.74 (0.09)	< 0.001
	Normalized lateral ventricle volume (SE) (mL)	14.7 (1.3)	21.9 (1.7)	0.001
	NAWM FA^b^ (SE)	0.336 (0.003)	0.323 (0.002)	< 0.001
	NAWM MD^b^ (SE)	0.811 (0.003)	0.820 (0.003)	0.026
	NAWM AD^b^ (SE)	1.118 (0.003)	1.116 (0.003)	0.638
	NAWM RD^b^ (SE)	0.657 (0.004)	0.671 (0.004)	0.009

Comparisons were performed by Fisher’s exact test (sex) and Mann–Whitney (age) tests. Age- and sex-adjusted linear models were performed for MRI features. ARR was estimated with a negative binomial regression. Classification of DMTs: MET = any preparation of interferon-beta, glatiramer acetate, teriflunomide, and dimethyl fumarate; HET = cyclophosphamide, mitoxantrone, cladribine, natalizumab, fingolimod, alemtuzumab, and ocrelizumab. AD = axial diffusivity; ARR = annualized relapse rate; 6m-CDW = 6-month confirmed disability worsening; CP = choroid plexus; DMT = disease-modifying therapy; EDSS = Expanded Disability Status Scale; FA = fractional anisotropy; HC = healthy controls; HET = high-efficacy treatment; IQR = interquartile range; MD = mean diffusivity; MET = moderate-efficacy treatment; mL = milliliter; No. = number; NA = normal appearing; NBV = normalized brain volume; NCV = normalized cortical volume; RD = radial diffusivity; SE = standard error; WM = white matter.

a T2-hyperintense white matter lesion volume was log-transformed.

b MD, AD, and RD are expressed in units of mm^2^/s × 10^−3^. FA is a dimensionless index.

Compared to HC, Ped-MS patients had significantly higher T2-hyperintense WM LV and CP volume (all *p* < 0.001), lower NBV, NCV and normalized thalamic volume (*p* ⩽ 0.023), as well as higher NAWM MD and RD (*p* ⩽ 0.026) and lower NAWM FA (*p* < 0.001). The median follow-up was 12.3 (interquartile range (IQR) = 10.1 to 14.3) years. Thirty-six (69%) experienced ⩾ 1 clinical relapse, 11 (21%) had a 6m-CDW event, and 18 (35%) had EDSS worsening, even though no significant overall EDSS changes were observed (median (interquartile range, IQR) = 0.0 (−1.0 to 1.0); median EDSS at baseline (IQR) = 1.5 (1.0 to 1.5) at follow-up = 1.0 (0.0 to 2.5) *p* = 0.785). No patients converted to secondary progressive MS. Of the 43 MET-treated patients, 25 (58%, 22 in interferon-beta and 3 in glatiramer acetate) switched to HETs for efficacy after a median of 3.45 years (IQR = 2.39 to 7.20). Although 15 patients switched to fingolimod during follow-up, none of the patients under 18 years of age received this treatment. HETs were initiated a median of 3.95 (IQR = 2.19 to 8.02) years from disease onset. Patients were treated for a median of 5.94 (IQR = 2.02 to 11.55) years with METs and of 4.98 (IQR = 0.00 to 9.71) years with HETs. The median proportions of follow-up time spent on METs and HETs were 53.4% (IQR = 15.4 to 100.0) and 46.6% (IQR = 0.0 to 84.6), respectively. During follow-up, treatment switches from MET to HET occurred exclusively for lack of efficacy, whereas no patient switched treatment category due to adverse events or non-compliance; no de-escalations from HET to MET were reported.

### Survival analysis of time to first relapse

**Figure 1.**
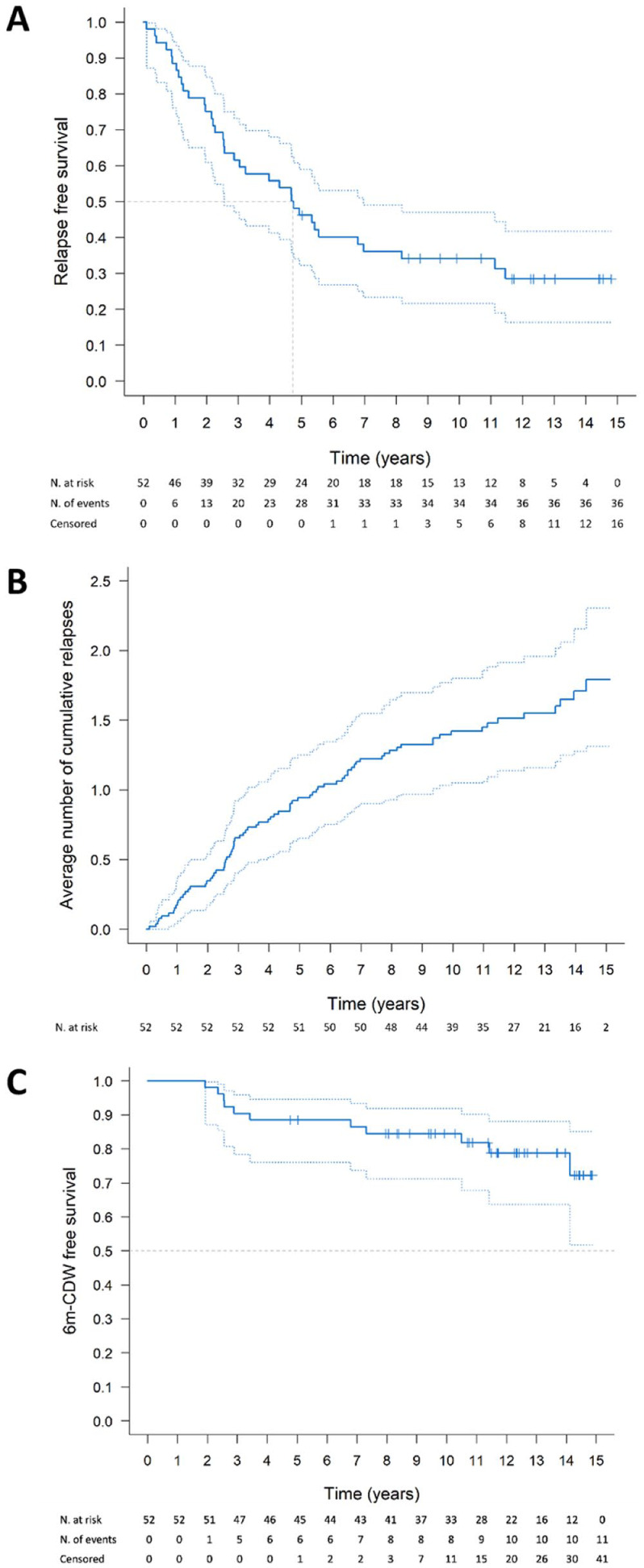
Relapse-free survival, relapse occurrence, and 6m-CDW-free survival in pediatric MS patients. Kaplan-Meier curves show the probability estimates of surviving relapses (a) and 6m-CDW events (c). The average cumulative number of relapses over the follow-up is represented in (b). Dotted lines are 95% pointwise confidence intervals. 6m-CDW = 6-month confirmed disability worsening; MS = multiple sclerosis.

The Kaplan–Meier estimate of relapse-free survival is represented in Figure 1(a). The median time from baseline to the first clinical relapse was 4.7 (95% confidence interval (CI) = 2.6 to 7.0) years. At multivariable analysis, higher infratentorial lesion number (hazard ratio (HR) = 1.09, 95% CI = 1.02 to 1.18), spinal cord lesion presence (HR = 2.45, 95% CI = 1.18 to 5.10), and lower thalamic volume (HR = 0.77, 95% CI = 0.60 to 0.99) were independently associated with shorter time to a first clinical relapse; higher CP volume (HR = 1.58, 95% CI = 0.93 to 2.68) was marginally associated. HET exposure was associated with lower relapse hazard (HR = 0.20, 95% CI = 0.06 to 0.68) (Table 2).

**Table 2.** Univariable and multivariable Cox regression models using time to first clinical relapse as the outcome in pediatric multiple sclerosis.

		Time to first clinical relapse			
		Univariable analysis		Multivariable analysis	
		HR (95% CI)	*p*	HR (95% CI)	*p*
Sex (male vs. female)		0.81 (0.41 to 1.61)	0.549		
Age at baseline		0.92 (0.81 to 1.04)	0.174		
Age at disease onset		0.94 (0.85 to 1.05)	0.271		
Disease duration		1.0 (0.85 to 1.18)	0.962		
EDSS score at baseline		1.63 (1.02 to 2.58)	0.039		
No. of previous relapses		1.18 (0.82 to 1.68)	0.369		
Previous treatment (yes vs. no)		0.61 (0.13 to 2.89)	0.530		
Treatment status (HET vs. MET)		0.60 (0.25 to 1.46)	0.259	0.20 (0.06 to 0.68)	0.010
Brain T2-hyperintense WM LV^a^		1.05 (1.01 to 1.10)	0.019		
No. of T2-hyperintense WM lesions	Periventricular	1.06 (1.01 to 1.11)	0.027		
	Juxtacortical	1.01 (1.00 to 1.02)	0.149		
	Infratentorial	1.10 (1.02 to 1.18)	0.009	1.09 (1.02 to 1.18)	0.016
	Deep WM	1.00 (0.96 to 1.06)	0.845		
No. of cortical lesions		1.27 (0.97 to 1.65)	0.077		
Cord lesions (yes vs. no)		2.13 (1.06 to 4.30)	0.035	2.45 (1.18 to 5.10)	0.017
NBV^b^		0.94 (0.88 to 1.00)	0.047		
NCV^b^		0.97 (0.89 to 1.05)	0.448		
Thalamic volume		0.72 (0.58 to 0.89)	0.003	0.77 (0.60 to 0.99)	0.046
Normalized CP volume		1.67 (1.04 to 2.69)	0.034	1.58 (0.93 to 2.68)	0.088
NAWM FA^c^		0.76 (0.59 to 0.97)	0.031		
NAWM MD^c^		1.13 (0.98 to 1.30)	0.091		
NAWM AD^c^		1.05 (0.92 to 1.20)	0.482		
NAWM RD^c^		1.14 (1.01 to 1.29)	0.041		

Hazard ratios (HR), related 95% CI, and *p*-values from extended Cox regression models are reported. Treatment status, modeled as a time-varying covariate, was included in all analyses. AD = axial diffusivity; CI = confidence interval; CP = choroid plexus; DMT = disease-modifying therapy; EDSS = Expanded Disability Status Scale; FA = fractional anisotropy; IQR = interquartile range; MD = mean diffusivity; mL = milliliter; NA = normal appearing; NBV = normalized brain volume; NCV = normalized cortical volume; RD = radial diffusivity; WM = white matter.

a HR is computed for a 10% increase in T2-hyperintense white matter lesion volume.

b HR is computed for a 10-unit increase in the predictor.

c HR is computed for a 0.01-unit increase in the predictor. MD, AD, and RD are expressed in units of mm^2^/s × 10^−3^. FA is a dimensionless index.

### Survival analysis of recurrent relapses

The estimated annualized relapse rate was 0.13 (95% CI = 0.10 to 0.17), significantly lower in HET than MET periods (rate ratio HET vs. MET = 0.37, 95% CI = 0.19 to 0.74, *p* = 0.005). These estimates reflect observed associations and do not imply causal effects. The average cumulative number of relapses experienced by patients over the follow-up is represented in Figure 1(b). At multivariable analysis, higher brain T2-hyperintense WM LV (HR = 1.04, 95% CI = 1.01 to 1.06) was associated with higher relapse risk during follow-up, while higher EDSS score (HR = 1.29, 95% CI = 0.96 to 1.73), spinal cord lesion presence (HR = 1.52, 95% CI = 0.95 to 2.44), and higher CP volume (HR = 1.35, 95% CI = 1.00 to 1.83) contributed marginally. A lower overall relapse risk was observed under HET exposure (HR = 0.21, 95% CI = 0.11 to 0.40) (Table 3).

**Table 3.** Univariable and multivariable Andersen–Gill models for recurrent relapse risk in pediatric multiple sclerosis.

		Recurrent relapses			
		Univariable analysis		Multivariable analysis	
		HR (95% CI)	*p*	HR (95% CI)	*p*
Sex (male vs. female)		0.90 (0.52 to 1.56)	0.712		
Age at baseline		0.93 (0.82 to 1.05)	0.256		
Age at disease onset		0.95 (0.87 to 1.04)	0.283		
Disease duration		1.01 (0.90 to 1.14)	0.864		
EDSS score at baseline		1.67 (1.32 to 2.11)	< 0.001	1.29 (0.96 to 1.73)	0.086
No. of previous relapses		1.04 (0.74 to 1.47)	0.813		
Previous treatment (yes vs. no)		1.30 (0.35 to 4.86)	0.693		
Treatment status (HET vs. MET)		0.43 (0.21 to 0.91)	0.027	0.21 (0.11 to 0.41)	< 0.001
Brain T2-hyperintense WM LV^a^		1.06 (1.02 to 1.09)	< 0.001	1.04 (1.01 to 1.06)	0.013
No. of T2-hyperintense WM lesions	Periventricular	1.08 (1.03 to 1.12)	< 0.001		
	Juxtacortical	1.02 (1.01 to 1.02)	< 0.001		
	Infratentorial	1.08 (1.03 to 1.13)	0.002		
	Deep WM	1.02 (0.98 to 1.06)	0.300		
No. of cortical lesions		1.17 (1.00 to 1.36)	0.051		
Cord lesions (yes vs. no)		1.85 (1.00 to 3.40)	0.049	1.52 (0.95 to 2.44)	0.081
NBV^b^		0.96 (0.92 to 1.01)	0.149		
NCV^b^		0.98 (0.90 to 1.06)	0.640		
Thalamic volume		0.83 (0.70 to 0.98)	0.030		
Normalized CP volume		1.46 (1.06 to 2.02)	0.019	1.35 (1.00 to 1.83)	0.054
NAWM FA^c^		0.80 (0.67 to 0.95)	0.012		
NAWM MD^c^		1.14 (1.02 to 1.29)	0.026		
NAWM AD^c^		1.07 (0.96 to 1.20)	0.213		
NAWM RD^c^		1.14 (1.03 to 1.26)	0.013		

Hazard ratios (HR), related 95% CI, and *p*-values from Andersen–Gill models are reported. Treatment status, modeled as a time-varying covariate, was included in all analyses. AD = axial diffusivity; CI = confidence interval; CP = choroid plexus; DMT = disease-modifying therapy; EDSS = Expanded Disability Status Scale; FA = fractional anisotropy; IQR = interquartile range; MD = mean diffusivity; mL = milliliter; NA = normal appearing; NBV = normalized brain volume; NCV = normalized cortical volume; RD = radial diffusivity; WM = white matter.

a HR is computed for a 10% increase in T2-hyperintense white matter lesion volume.

b HR is computed for a 10-unit increase in the predictor.

c HR is computed a 0.01-unit increase in the predictor. MD, AD, and RD are expressed in units of mm^2^/s × 10^−3^. FA is a dimensionless index.

### Survival analysis of time to 6m-CDW event

The Kaplan-Meier survival curve is represented in Figure 1(c). At 5 and 10 years of follow-up, 88% (95% CI = 76 to 95) and 84% (95% CI = 71 to 92) of Ped-MS patients remained free from 6m-CDW. Younger age (HR = 0.82, 95% CI = 0.68 to 0.98) and lower NAWM FA (HR = 0.67, 95% CI = 0.51 to 0.88) were independently associated with shorter time to a 6m-CDW event (Table 4).

**Table 4.** Univariable and multivariable Cox regression models using time to 6m-CDW event as outcome in pediatric multiple sclerosis.

		6m-CDW			
		Univariable analysis		Multivariable analysis	
		HR (95% CI)	*p*	HR (95% CI)	*p*
Sex (male vs. female)		0.94 (0.27 to 3.23)	0.919		
Age at baseline		0.75 (0.60 to 0.94)	0.014	0.82 (0.68 to 0.98)	0.029
Age at disease onset		0.89 (0.72 to 1.10)	0.291		
Disease duration		0.82 (0.56 to 1.19)	0.292		
EDSS score at baseline		1.11 (0.52 to 2.38)	0.783		
No. of previous relapses		0.92 (0.52 to 1.64)	0.783		
Previous treatment (yes vs. no)		0.96 (0.11 to 8.22)	0.970		
Treatment status (HET vs. MET)		1.16 (0.33 to 4.03)	0.818	0.75 (0.18 to 30.97)	0.690
Brain T2-hyperintense WM LV^a^		1.02 (0.95 to 1.08)	0.623		
No. of T2-hyperintense WM lesions	Periventricular	0.99 (0.90 to 1.09)	0.852		
	Juxtacortical	1.00 (0.98 to 1.02)	0.693		
	Infratentorial	1.09 (0.99 to 1.20)	0.069		
	Deep WM	0.96 (0.85 to 1.07)	0.460		
No. of cortical lesions		1.20 (0.81 to 1.78)	0.372		
Cord lesions (yes vs. no)		0.94 (0.28 to 3.16)	0.925		
NBV^b^		0.99 (0.88 to 1.11)	0.843		
NCV^b^		1.10 (0.94 to 1.29)	0.226		
Thalamic volume		0.84 (0.60 to 1.17)	0.308		
Normalized CP volume		0.48 (0.18 to 1.25)	0.132		
NAWM FA^c^		0.62 (0.46 to 0.85)	0.003	0.67 (0.51 to 0.88)	0.004
NAWM MD^c^		1.30 (1.05 to 1.60)	0.017		
NAWM AD^c^		1.17 (0.89 to 1.54)	0.273		
NAWM RD^c^		1.27 (1.06 to 1.52)	0.009		

Hazard ratios (HR), related 95% CI, and *p*-values from extended Cox regression models are reported. Treatment status, modeled as a time-varying covariate, was included in all analyses. 6m-CDW = 6-month confirmed disability worsening; AD = axial diffusivity; CI = confidence interval; CP = choroid plexus; DMT = disease-modifying therapy; EDSS Expanded Disability Status Scale; FA = fractional anisotropy; HET = high-efficacy treatment; IQR = interquartile range; MD = mean diffusivity; mL = milliliter; MET = moderate-efficacy treatment; NA = normal appearing; NBV = normalized brain volume; NCV = normalized cortical volume; RD = radial diffusivity; WM = white matter.

a HR is computed for a 10% increase in T2-hyperintense white matter lesion volume.

b HR is computed for a 10-unit increase in the predictor.

c HR is computed for a 0.01-unit increase in the predictor. MD, AD, and RD are expressed in units of mm^2^/s × 10^−3^. FA is a dimensionless index.

### Survival analysis of EDSS worsening at follow-up

Lower NAWM FA was associated with greater EDSS worsening at last follow-up (β = −0.26, 95% CI = −0.50 to −0.03) (Table 5).

**Table 5.** Univariable and multivariable associations of demographic, clinical, and MRI variables with EDSS worsening at last follow-up in pediatric multiple sclerosis.

		EDSS worsening at follow-up			
		Univariable analysis		Multivariable analysis	
		β (95% CI)	*p*	β (95% CI)	*p*
Sex (male vs. female)		-0.25 (-0.98 to 0.48)	0.488		
Age at baseline		-0.10 (-0.28 to 0.08)	0.254		
Age at disease onset		-0.03 (-0.17 to 0.10)	0.628		
Disease duration		-0.04 (-0.21 to 0.13)	0.653		
EDSS score at baseline		-0.37 (-0.92 to 0.19)	0.190		
No. of previous relapses		0.15 (-0.18 to 0.49)	0.366		
Previous treatment (yes vs. no)		-0.41 (-1.80 to 0.98)	0.553		
Treatment at baseline (HET vs. MET)		0.20 (-0.81 to 1.21)	0.691	-0.27 (-1.33 to 0.78)	0.606
Switch to HET line treatment		0.16 (-0.62 to 0.94)	0.681	0.10 (-0.65 to 0.85)	0.791
Brain T2-hyperintense WM LV^a^		0.01 (-0.03 to 0.05)	0.475		
No. of T2-hyperintense WM lesions	Periventricular	0.02 (-0.04 to 0.08)	0.529		
	Juxtacortical	0.00 (-0.01 to 0.02)	0.651		
	Infratentorial	0.07 (0.00 to 0.14)	0.047		
	Deep WM	-0.00 (-0.07 to 0.06)	0.938		
No. of cortical lesions		0.22 (-0.05 to 0.50)	0.112		
Cord lesions (yes vs. no)		0.30 (-0.40 to 1.01)	0.391		
NBV^b^		-0.03 (-0.09 to 0.03)	0.341		
NCV^b^		0.01 (-0.08 to 0.10)	0.767		
Thalamic volume		-0.20 (-0.42 to 0.02)	0.068		
Normalized CP volume		0.11 (-0.37 to 0.58)	0.657		
NAWM FA^c^		-0.26 (-0.50 to -0.03)	0.029	-0.26 (-0.50 to -0.03)	0.029
NAWM MD^c^		0.06 (-0.10 to 0.22)	0.452		
NAWM AD^c^		-0.07 (-0.25 to 0.11)	0.461		
NAWM RD^c^		0.09 (-0.05 to 0.23)	0.216		

Beta coefficients (β), related 95% CI, and *p*-values from multiple linear regression models are reported. Analyses are adjusted for follow-up duration, baseline treatment, and treatment change. AD = axial diffusivity; CI = confidence interval; CP = choroid plexus; DMT = disease-modifying therapy; EDSS = Expanded Disability Status Scale; FA = fractional anisotropy; HET = high-efficacy treatment; IQR = interquartile range; MD = mean diffusivity; MET = moderate efficacy treatment; mL = milliliter; NA = normal appearing; NBV = normalized brain volume; NCV = normalized cortical volume; RD = radial diffusivity; WM = white matter.

a β regression coefficient quantifies the estimated mean EDSS worsening for a 10% increase in T2-hyperintense white matter lesion volume.

b β regression coefficient quantifies the estimated mean EDSS worsening for a 10-unit increase in the predictor.

c β regression coefficient quantifies the estimated mean EDSS worsening for a 0.01-unit increase in the predictor. MD, AD, and RD are expressed in units of mm^2^/s × 10^−3^. FA is a dimensionless index.

## Discussion

This longitudinal study aimed to improve the understanding of the correlates associated with 12-year clinical outcome in Ped-MS patients by integrating advanced MRI with clinical and routine MRI features to possibly optimize personalized treatment and improve long-term outcomes of these patients.

Half of patients experienced a clinical relapse within the first 5 years from baseline, consistent with earlier studies reporting higher relapse rates during the first years of disease.^21^

Infratentorial and spinal cord lesions and lower thalamic volume were associated with shorter time to first relapse, whereas higher CP volume showed a marginal association. Spinal cord lesions and CP enlargement also tended to relate to higher overall relapse risk during follow-up.

Although supratentorial WM lesion distribution resembles adult MS,^22^ Ped-MS patients show a preferential infratentorial involvement.^22–24^ This pattern likely reflects the maturational caudo-rostral gradient of myelination^25^ with myelin-rich infratentorial and spinal regions being more vulnerable, and aligns with previous studies in adult MS, showing the importance of these regions in predicting future relapses.^11,26^

Compared with HC, our pediatric cohort showed higher CP volume, which in turn displayed a marginal association with higher relapse risk. Previous studies performed in Ped-MS patients showed no associations between CP enlargement and EDSS score at baseline^16,17^ nor with the occurrence of new relapses over a 1-year follow-up.^17^ This discrepancy with previous studies likely reflects differences in sample size, clinical features (e.g. a shorter disease duration in our study), follow-up duration (12 years vs. 1 year in the previous study), and methodological approaches (e.g. CP segmentation (monocentric 3.0 T scanner vs. multicentric 1.5 T scanners) and normalization strategies). Taken together, these findings suggest that CP enlargement may represent an early marker of neuroinflammation in MS, not correlating with short-term clinical outcomes, but rather with the cumulative relapse risk over long-term. This aligns with evidence from adult MS, where CP enlargement has been associated with relapse rate over 4 years of follow-up.^18^ Accordingly, integration of CP volumetry could provide relevant prognostic information, particularly in patients close to disease onset, when clinical relapses and MRI activity are most pronounced.

Thalamic atrophy is associated with shorter time to relapse. The thalamus is among the earliest GM structures affected in MS.^27^ Mechanisms of thalamic neurodegeneration include intrinsic MS-related pathology and CSF-mediated toxicity.^28^ Moreover, retrograde Wallerian degeneration from WM lesions^29^ likely drives early thalamic atrophy, especially in patients with a high burden of T2-hyperintense WM lesions and inflammatory activity,^30^ consistent with previous studies in adult MS showing early thalamic involvement^27^ predicting 5-year clinical progression.^13^

Higher brain T2-hyperintense WM LV was significantly related to higher overall relapse risk during follow-up, whereas baseline EDSS showed a marginal association. Both measures reflect greater disease burden, typical of patients with a more active disease course and with a higher likelihood of experiencing clinical exacerbations over time. In addition, they are both influenced by infratentorial and spinal involvement, which aligns with the topography of lesions associated with relapses found in our study.

HET exposure was associated with a longer time to first clinical relapse and lower overall relapse risk. Our analyses cannot establish causal effects, and the relatively small number of patients receiving HETs limits the robustness of conclusions. Nevertheless, our result aligns with recent studies^7,8,31^ showing that the early beginning of HET in Ped-MS patients is associated with more effective relapse control compared with METs.

Studies from multicenter cohorts and national registries^31^ consistently report lower relapse rates and better relapse control in children receiving newer HETs than in those treated with injectables and METs.

Moreover, although more than half of the follow-up time in our cohort was spent on METs, a substantial proportion of patients required escalation to HETs after a median time of 3.45 years. This suggests that some patients lacking baseline negative prognostic factors may initially remain stable on platform therapies. Nevertheless, the majority required escalation, highlighting the highly inflammatory nature of Ped-MS. Overall, our findings are consistent with the potential benefits of early HET exposure, while careful monitoring may allow a risk-adapted approach in selected cases.

At follow-up, 21% of patients had 6m-CDW, and 35% showed EDSS worsening. Younger age and lower FA NAWM are independently associated with shorter time to a first 6m-CDW event, with NAWM FA also associating with long-term EDSS worsening. These findings seem in contrast with previous studies showing that older age at onset associates with disability progression in Ped-MS patients.^6–8^ Explanations may include diagnostic delays in younger patients since MS symptoms in these patients can sometimes be mistaken for other conditions, thus delaying diagnosis and DMT initiation. Moreover, younger patients may have been prescribed METs due to safety concerns, thus influencing the rate of disability worsening. However, the therapeutic scenario has significantly changed in recent years, with the introduction of HETs from disease onset, even in younger children.^31^ Finally, a proportion of younger patients included in previous studies, particularly those performed before systematic testing for MOG antibodies, may have had MOGAD rather than MS, potentially contributing to discordant findings regarding long-term outcomes.

Interestingly, NAWM microstructural damage is associated with EDSS worsening, suggesting worse outcome in those patients having diffuse WM abnormalities. Several studies have shown NAWM abnormalities in Ped-MS, both in terms of maturational changes and loss of tissue integrity.^32,33^ In adult MS, pathological-MRI studies have revealed axonal injury, gliosis, demyelination, and widespread microglia activation in NAWM, although less severe than in focal WM lesions,^34^ which contribute to clinical worsening.^15,35^ DT-MRI measures can detect these early abnormalities, which precede irreversible atrophy, and may serve as a sensitive biomarker to guide treatment intensification before neurodegeneration becomes irreversible.

Cortical lesion number was not associated with 6m-CDW in our Ped-MS cohort. This contrasts with adult studies, where cortical lesions are associated with disability worsening.^10^ However, Ped-MS patients typically exhibit a lower cortical lesion burden than adults, especially in early disease stages.^36^ Moreover, despite the use of DIR sequences, the sensitivity of MRI for detecting cortical lesions remains limited, particularly in children, due to motion artifacts and smaller brain volumes. Finally, the pathological relevance of cortical lesions in Ped-MS may differ from that in adult MS, possibly due to greater neuroplasticity and repair mechanisms in younger brains.^3^ These aspects may attenuate the clinical impact of cortical demyelination in these patients.

This study holds some limitations. First, dropout (25% in our pediatric cohort) is inherent to long-term observational cohorts and in line with previous studies with a similar follow-up.^37^ However, attrition was primarily related to changes in the MS center or relocation abroad, rather than clinical worsening, reducing the risk of bias. We acknowledge that this limitation could still impact the generalizability of our findings. Future studies with larger samples are warranted to confirm and extend our results. Second, spinal cord MRI was not acquired under a standardized research protocol but as part of clinical care, and the timing of spinal MRI was not fully standardized relative to baseline brain MRI. In addition, only lesion presence/absence was available, without quantitative data on lesion burden or spinal cord atrophy, which may limit the precision of our analyses. Nevertheless, spinal MRI was systematically performed in all patients as part of the diagnostic work-up, regardless of onset type or spinal cord symptoms, and in some cases was repeated following the occurrence of clinical symptoms. These examinations were consistently obtained close to the baseline brain MRI, as all patients were enrolled within the first three years of disease. Future longitudinal studies should implement standardized spinal cord MRI protocols and quantitative assessments to clarify their contribution to long-term clinical outcomes.

Third, we did not evaluate gadolinium-enhancing lesions at baseline. However, our patients had to be relapse- and steroid-free for at least 1 month prior to clinical and MRI assessment. Fourth, cognitive outcomes were not included. Cognitive impairment is increasingly recognized as a major burden in Ped-MS and is expected to correlate with cortical and thalamic atrophy, diffuse WM damage, and glymphatic system impairment. Future research should incorporate standardized cognitive measures alongside imaging to provide a more comprehensive view of disease impact. Fifth, although conventional MRI confirmed prior observations in Ped-MS, advanced MRI provided novel insights.

Finally, given the observational design, non-randomized treatment allocation, and potential residual confounding by indication, our models identified associations rather than causal effects. Future studies should validate and extend our findings also integrating standardized cognitive endpoints in larger, more heterogeneous pediatric cohorts, accounting for different treatment strategies, including newer imaging biomarkers such as chronic active lesions and spinal cord atrophy, and applying causal-inference methods, to support the development of prognostic models.

Our findings indicate that both focal and diffuse inflammatory disease activity were associated with long-term disease outcomes in pediatric patients with clinically definite MS. HET exposure was associated with a longer time to first relapse and a lower overall relapse risk. These associations align with evidence supporting early use of highly effective therapies and underscore the value of advanced MRI markers for monitoring and treatment planning in Ped-MS.

## Supplemental Material

sj-docx-1-msj-10.1177_13524585251408639 – Supplemental material for Correlates of long-term clinical outcomes in pediatric multiple sclerosis: A 12-year study Supplemental material, sj-docx-1-msj-10.1177_13524585251408639 for Correlates of long-term clinical outcomes in pediatric multiple sclerosis: A 12-year study by Monica Margoni, Alessandro Meani, Elisabetta Pagani, Paolo Preziosa, Lucia Moiola, Mattia Pozzato, Eleonora Tavazzi, Flavia Mattioli, Valentina Torri Clerici, Massimo Filippi and Maria A Rocca in Multiple Sclerosis Journal

sj-docx-2-msj-10.1177_13524585251408639 – Supplemental material for Correlates of long-term clinical outcomes in pediatric multiple sclerosis: A 12-year study Supplemental material, sj-docx-2-msj-10.1177_13524585251408639 for Correlates of long-term clinical outcomes in pediatric multiple sclerosis: A 12-year study by Monica Margoni, Alessandro Meani, Elisabetta Pagani, Paolo Preziosa, Lucia Moiola, Mattia Pozzato, Eleonora Tavazzi, Flavia Mattioli, Valentina Torri Clerici, Massimo Filippi and Maria A Rocca in Multiple Sclerosis Journal
