# Supplementary material for: Correlates of long-term clinical outcomes in pediatric multiple sclerosis: A 12-year study
Source: Mult Scler. 2026 Jan 26;32(6):585–97. doi: 10.1177/13524585251408639 (PMC13168603; doi:10.1177/13524585251408639)
Supplement: sj-docx-1-msj-10.1177_13524585251408639 – Supplemental material for Correlates of long-term clinical outcomes in pediatric multiple sclerosis: A 12-year study [file sj-docx-1-msj-10.1177_13524585251408639.docx]

MS Journal Appendix for MRI methodology

| **Hardware** | |
| --- | --- |
| Field strength | 3.0 T |
| Manufacturer | Philips Medical Systems |
| Model | Intera |
| Coil type  (e.g. head, surface) | head coil |
| Number of coil channels | 8 |

| **Acquisition sequence** | | |
| --- | --- | --- |
| Type  (e.g. FLAIR, DIR, DTI, fMRI) | dual-echo turbo spin echo (TSE) | |
| Acquisition time | 7.21 min | |
| Orientation | axial | |
| Alignment  (e.g. anterior commissure/poster commissure line | Inferior border of the corpus callosum | |
| Voxel size | 0.94x0.94x3 mm | |
| TR | 2599 ms | |
| TE | 16,80 ms | |
| TI | - | |
| Flip angle | 90 | |
| NEX | 1 | |
| Field of view | 240x240 mm | |
| Matrix size | 256x256 | |
| Parallel imaging | Yes | No |
| If used, parallel imaging method:  (e.g. SENSE, GRAPPA) | - | |
| Cardiac gating | Yes | No |
| If used, cardiac gating method:  (e.g. PPU or ECG) |  | |
| Contrast enhancement | Yes | No |
| **Acquisition sequence** | | |
| If used, provide name of contrast agent, dose and timing of scan post-contrast administration |  | |
| Other parameters:  Echo train length | 6 | |

| **Acquisition sequence** | | |
| --- | --- | --- |
| Type  (e.g. FLAIR, DIR, DTI, fMRI) | 3D *T*_1_ | |
| Acquisition time | 6.34 min | |
| Orientation | Axial | |
| Alignment  (e.g. anterior commissure/poster commissure line) | Inferior border of the corpus callosum | |
| Voxel size | 0.9x0.9x0.8 mm | |
| TR | 75 ms | |
| TE | 4.6 ms | |
| TI | - | |
| Flip angle | 30° | |
| NEX | 1 | |
| Field of view | 230x184 mm | |
| Matrix size | 256x204 | |
| Parallel imaging | Yes | No |
| If used, parallel imaging method:  (e.g. SENSE, GRAPPA) | SENSE | |
| Cardiac gating | Yes | No |
| If used, cardiac gating method:  (e.g. PPU or ECG) |  | |
| Contrast enhancement | Yes | No |

| **Acquisition sequence** | | |
| --- | --- | --- |
| Type  (e.g. FLAIR, DIR, DTI, fMRI) | DIR | |
| Acquisition time | 9 min | |
| Orientation | Axial | |
| Alignment  (e.g. anterior commissure/poster commissure line) | Inferior border of the corpus callosum | |
| Voxel size | 0.9x0.9x3 mm | |
| TR | 18146 ms | |
| TE | 125 ms | |
| TI | 3000/100 | |
| Flip angle | 180° | |
| NEX | 2 | |
| Field of view | 240x180 mm | |
| Matrix size | 256x189 | |
| Parallel imaging | Yes | No |
| If used, parallel imaging method:  (e.g. SENSE, GRAPPA) |  | |
| Cardiac gating | Yes | No |
| If used, cardiac gating method:  (e.g. PPU or ECG) |  | |
| Contrast enhancement | Yes | No |

| **Acquisition sequence** | | |
| --- | --- | --- |
| Type  (e.g. FLAIR, DIR, DTI, fMRI) | DTI | |
| Acquisition time | 11 min | |
| Orientation | Axial | |
| Alignment  (e.g. anterior commissure/poster commissure line) | Inferior border of the corpus callosum | |
| Voxel size | 2.14x2.7x1.3 mm | |
| TR | 8779 ms | |
| TE | 58 ms | |
| TI | - | |
| Flip angle | 90° | |
| NEX | 1 | |
| Field of view | 240x228 mm | |
| Matrix size | 112x88 | |
| Parallel imaging | Yes | No |
| If used, parallel imaging method:  (e.g. SENSE, GRAPPA) | SENSE | |
| Cardiac gating | Yes | No |
| If used, cardiac gating method:  (e.g. PPU or ECG) |  | |
| Contrast enhancement | Yes | No |
| Other parameters: | 35 diffusion weighted directions, b=900 s/mm^2^ | |

| **Image analysis methods and outputs** | |
| --- | --- |
| ***Brain lesions*** | |
| Type  (e.g. Gd-enhancing, T2-hyperintense, T1-hypointense) | *T*_2_-hyperintense |
| Analysis method | local thresholding segmentation technique |
| Analysis software | Jim 8.0 (Xinapse Systems) |
| Output measure  (e.g. count or volume [ml]) | Volume [ml] |
| ***Brain tissue volumes*** | |
| Type  (e.g. whole brain, grey matter, white matter, spinal cord) | Whole brain, cortical gray matter, thalami and ventricles |
| Analysis method | Fully automated tissue segmentation |
| Analysis software | FSL-SIENAx, FIRST, Jim 8.0 |
| Output measure  (e.g. absolute tissue volume in ml, tissue volume as a fraction of intracranial volume, percentage change in tissue volumes) | Tissue volume [ml] normalized for head size |
| ***Lesion topography*** | |
| Type  (e.g. Gd-enhancing, T2-hyperintense, T1-hypointense) | *T*_2_-hyperintense |
| Analysis method | Identification of infratentorial, periventicular, juxtacortical and deep WM lesions. |
| Analysis software | Pipeline using tools from the FSL library and matlab routines |
| Output measure  (e.g. absolute tissue volume in ml, tissue volume as a fraction of intracranial volume, percentage change in tissue volumes) | Lesion topography |
| ***Tissue measures*** | |
| Type  (e.g. MTR, DTI, T1-RT, T2-RT, T2*, T2’, ^1^H-MRS, perfusion, Na) | DTI |
| Analysis method | Diffusion tensor analysis and derived metrics within the normal appearing white matter |
| Analysis software | FSL library |
| Output measure | Average FA, MD, AD and RD |
| ***Other MRI measures (e.g. functional MRI)*** | |
| Type  (e.g. whole brain, grey matter, white matter, spinal cord, normal-appearing grey matter or white matter) | Choroid plexus of lateral ventricles |
| Analysis method | Manual segmentation |
| Analysis software | Jim 8 (Xinapse Systems) |
| Output measure | volume [ml] normalized for head size |
