# Supplementary material for: Correlates of long-term clinical outcomes in pediatric multiple sclerosis: A 12-year study
Source: Mult Scler. 2026 Jan 26;32(6):585–97. doi: 10.1177/13524585251408639 (PMC13168603; doi:10.1177/13524585251408639)
Supplement: sj-docx-2-msj-10.1177_13524585251408639 – Supplemental material for Correlates of long-term clinical outcomes in pediatric multiple sclerosis: A 12-year study [file sj-docx-2-msj-10.1177_13524585251408639.docx]

**SUPPLEMENTARY MATERIAL**

**Supplementary Methods**

MRI acquisition. Using the same 3.0 T Philips Intera MR scanner with 8-channel head coil (Philips Medical System), the following sequences of the brain were acquired from all subjects during a single session at baseline: 1) dual-echo turbo spin echo (repetition time [TR]=2599 milliseconds [msec]; echo time [TE]=16-80 msec; flip angle=90°; matrix=256×256; field of view [FOV]=240 mm^2^; echo train length [ETL]=6; 44 contiguous axial slices, 3-mm thick); 2) three-dimensional (3D) T1-weighted fast field echo (TR=25 msec; TE=4.6 msec; flip angle=30°; matrix=256×256; FOV=230 mm^2^; 220 contiguous axial slices, 0.8-mm thick); 3) 3D double inversion recovery (DIR) (TR/TE=18,000/125 msec; inversion time [TI]=3000 msec; delay=100 msec; ETL=27; matrix=256×256; FOV=240×240 mm^2^; 44 axial 3-mm-thick slices); 4) pulsed-gradient spin echo echo-planar imaging (TR/TE=8775/58 msec, matrix=112×88, FOV=240×231 mm^2^, 55 contiguous, 2.3-mm thick axial slices) with SENSE (acceleration factor=2) and diffusion gradients applied in 35 non-collinear directions. Two optimized b factors were used for acquiring diffusion weighted images (b1=0, b2=900 s/mm^2^).

Lesional and volumetric MRI analysis. For lesion topography definition, we first identified each lesion using a routine in Matlab that labels connected components; then we transformed the relevant masks obtained from FSL-SIENAx2 software (cortical gray matter [GM], ventricles, cerebellum and brainstem) onto the space of lesion segmentation and identified infratentorial lesions (the sum of cerebellum and brainstem lesions); after the application of a 3-mm sphere kernel we identified periventricular and juxtacortical lesions as those touching the corresponding dilated masks; all the other lesions were coded as deep white matter [WM].

DT MRI analysis. Dual-echo and 3D T1-weighted sequences were separately co-registered to the distortion-free b=0 image, and the calculated transformations were applied to the binary masks of T2-hyperintense WM lesions and of WM previously obtained from SIENAx2. To evaluate microstructural tissue damage, fractional anisotropy (FA), mean (MD), axial (AD), and radial diffusivity (RD) values within the normal-appearing (NA) WM were derived after removing T2-hyperintense WM lesions from the WM mask.
